# An Advanced Deep Attention Collaborative Mechanism for Secure Educational Email Services

**DOI:** 10.1155/2022/3150626

**Published:** 2022-04-26

**Authors:** Yanfang Chen, Yongzhao Yang

**Affiliations:** Zhengzhou Preschool Education College, Zhengzhou 450000, China

## Abstract

The COVID-19 crisis has once again highlighted the vulnerabilities of some critical areas in cyberspace, especially in the field of education, as distance learning and social distance have increased their dependence on digital technologies and connectivity. Many recent cyberattacks on e-learning systems, educational content services, and trainee management systems have created severe demands for specialized technological solutions to protect the security of modern training methods. Email is one of the most critical technologies of educational organizations that are attacked daily by spam, phishing campaigns, and all kinds of malicious programs. Considering the efforts made by the global research community to ensure educational processes, this study presents an advanced deep attention collaborative filter for secure academic email services. It is a specialized application of intelligent techniques that, for the first time, examines and models the problem of spam as a system of graphs where collaborative referral systems undertake the processing and analysis of direct and indirect social information to detect and categorize spam emails. In this study, nonnegative matrix factorization (NMF) is applied to the social graph adjacent table to place users in one (or more) overlapping communities. Also, using a deep attention mechanism, it becomes personalized for each user. At the same time, with the introduction of exponential random graph models (ERGMs) in the process of factorization, local dependencies are significantly mitigated to achieve the revelation of malicious communities. This methodology is being tested successfully in implementing mail protection systems for educational organizations. According to the findings, the proposed algorithm outperforms all other compared algorithms in every metric tested.

## 1. Introduction

During the coronavirus pandemic, the rapid and violent digital transformation is called upon to deal with a massive wave of sophisticated and persistent cyberattacks related to the new reality [1–4]. This new reality is becoming particularly evident in education, which has become one of the ideal targets for digital attacks, as distance learning has become a necessity for billions of students worldwide [5].

In particular, advanced phishing campaigns are constantly evolving, using e-learning and distance education, access to education services, and educational content management systems as a theme. Schools and universities have switched to large-scale e-learning platforms, often without knowing crucial privacy issues [6]. Students in distance learning programs are also attacked daily. Furthermore, the rate of ransomware attacks is growing exponentially, with specialized cybercriminals first extracting large amounts of sensitive personal data before encrypting the educational databases of an institution or training organization. They then threaten to publish these data unless a ransom is paid, putting additional pressure on the organizations called upon to meet the criminals' demands in question [7].

The recipients are targeted by phishing attacks, which imitate the login portals of universities to steal credentials, etc. In most cases, these scams are related to the email of educational organizations, where the fundamental security flaws can be easily bypassed. In most phishing campaigns targeting educational organizations, the attack begins with an email that supposedly contains information about the institutions' instructions on complying with the COVID-19 protocols set by the relevant ministries, with instructions for course changes, grading, hypothetical links to digital classrooms, etc. Misleading spam emails encourage the recipient to click on an attached HTML file, which leads them to a fake login page similar to the teacher's login page, with disastrous results. These pages look very convincing, and the URLs use a similar naming pattern that includes the top-level domain of the relevant educational organizations [8, 9].

The most common and widely used machine learning technique for protection against related threats and the intelligent classification of spam emails is based on the use of measures that are based on distance. These methods are considered supervised learning methods, which presuppose that the whole set includes the input data and the desired categorization for each element [10, 11].

To categorize each new element in a class, it is necessary to calculate its distance from each part of the training set, finally considering only the assignments closest to it. These methods are based on analogy and not on producing a generalization model. Thus, there is no training stage, and no model is created until a new observation needs to be categorized. For this reason, the relevant categorizers are also called lazy classifiers [12]. Also, the specific methods for classifying a new observation must compare it with available observations of the training set, which requires storing all or at least part of the training data.

Although these methods can significantly achieve the complexity of detecting complex dependencies between the variables that make up the problem, they are relatively simple to implement and use and can achieve high classification performance [12, 13]. Many comparisons between observations require very effective indexing techniques. Their categorization of new observations takes longer and requires high availability of computing resources. Furthermore, the classification results are sensitive to the local characteristics of the data, the existence of insignificant input variables, and the number of categorization observations, increasing the risk of over-adaptation. We propose an innovative methodology to use a personalized attention mechanism to overcome the relevant obstacles.

The study is organized as follows: Section 2 provides an overview of the various relevant approaches that have been identified in the literature.  Section 3 presents the proposed methodology. The scenarios and results are presented in Section 4. Finally, a summary of the findings and a list of potential future research directions are concluded in Section 5.

## 2. Literature Review

The recent literature concerning the field of detection mechanisms for email services is as follows.

Abdullahi and Kaya [14] suggested a deep learning system for detecting phishing in emails and messages. On both email and SMS collections, they utilized ML algorithms such as SVM classifier, multinomial naive Bayes, decision tree, random forest, logistic regression, and dense neural network. They used existing assessment criteria to evaluate the classifiers on the datasets they employed. The analysis was carried out using coding techniques and the TensorFlow technology, and the outcome revealed that dense neural networks outperform deep learning classifications in identifying phishing attempts in all the samples. The suggested strategy outperformed traditional machine learning algorithms on real datasets.

Fang et al. [15] employed an algorithm dubbed THEMIS to identify suspicious emails. They began by analyzing the email layout and then recommended their scheme, which was used to model emails at the email header, body, character, and word level simultaneously, using an improved recurrent convolutional neural network (RCNN) model with multilevel vectors and attention mechanism. This approach was used in both the header and the model's body, causing it to pay greater attention to the more critical information between them. They utilized an imbalanced dataset with actual ratios of phishing and genuine emails to perform tests and assess THEMIS achieving overall accuracy high levels, according to the testing data. In the meanwhile, the false-positive rate was low. The filter's accuracy and low FPR help detect phishing emails with high likelihood, while benevolent emails were filtered out as little as possible. They aim to improve their model for identifying phishing emails that do not have a header and simply have content.

Phomkeona and Okamura [16] proposed a method for categorizing and diagnosing zero-day malicious emails based on data gathered from the email header and content. They merged it with dynamic analytic data as a collection of 27 features, including machine translation detection, risk word detection, and other characteristics, using numerous application programming interfaces. To teach and evaluate the system, four distinct language email datasets were employed to replicate real-world diversity and zero-day harmful email attack scenarios. They achieved a reasonable detection rate for both zero-day malicious email types and regular spam. They stated that by adding new contaminated spam datasets to train the algorithm and utilizing a translation API to boost accuracy, their model could be improved.

Kaddoura et al. [17] applied deep learning methods to identify link-less emails, and they proposed a spam email detection system based on FFNN. Different settings were used to optimize hyperparameters. The Enron dataset was preprocessed, and two feature extraction algorithms were used. On the Enron dataset, their model was tested to classify emails as spam or regular. This approach is compared to the BERT dataset. They also looked at numerous variants of its design in terms of layer count, the number of neurons per layer, and the number of neurons per input layer and calculated the *F*1 score for each one. Precision, recall, and accuracy were calculated to illustrate the approach's success.

Asudani et al. [18] investigated the efficiency of a pretrained embedding model for email categorization using deep learning filters such as the long short-term memory and convolutional neural network models. They employed pretrained word embedding using global vectors (GloVe) and Bidirectional Encoder Representations Transformers (BERT) to discover links between words, which helped them categorize messages into relevant categories using machine learning models. They concluded that word embedding models boost the accuracy of the email classifier. The experimentation used two benchmark datasets: SpamAssassin and Enron. GloVe embedding achieved quicker execution and improved performance on massive datasets, according to the research. Traditional machine learning methods categorize an email as benevolent or spam, and the CNN model with GloVe embedding yields somewhat greater accuracy than the model with BERT embedding.

Based on imperative phrases, Ali [19] proposed a framework for categorizing email content into three categories: order/command, request, and general. For email categorization, this study employed Word2Vec to convert words into vectors and two deep learning algorithms, namely convolutional neural networks and recurrent neural networks. They experimented using a sufficient email data collection obtained from a personal Gmail account and Enron. A random 10% of the dataset is used to test the machine, while 90% of the dataset is used for training the machine. Increases in the training dataset ratio result in enhanced algorithm accuracy, according to these trials. The experimental results reveal that RNN outperformed CNN in terms of accuracy. They also compared their approaches to the previously used method fuzzy ANN and found that their suggested methods CNN and RNN outperformed fuzzy ANN. They want to test the model on larger datasets since they think that combining many models and utilizing a hybrid method will increase accuracy.

In the research presented so far, the identification of spam and utilizing machine learning do not include the social information extracted from collaborative filtering algorithms, neither around users nor around objects. However, social information is perhaps the main springboard for the early suppression of spam and its scams. It is imperative to integrate it into forecasting systems. One of them is to find user communities based on the links (e.g., friendship and trust) they have in the social network. These communities can then be used to generate suggestions—predictions, which will reveal how to spread the unwanted content and, respectively, to identify potentially infected nodes that are bots in botnets. In general, the placement of users in “neighborhoods” on the social network is based on NMF techniques, where each user is not considered to belong exclusively to one neighborhood.

In the same way that a user does not rate items from a single category, so his social contacts do not fall into a single class (e.g., some may be coworkers, some friends, and some may belong in both groups mentioned above). On the contrary, they may belong to more, with a different percentage of participation in each. Therefore, the discovery and exploitation of these distinct groupings are expected to improve the production of recommendations—forecasts [20, 21].

NMF will be applied to the social graph adjacent table to place users in one (or more) overlapping communities in this research. Also, the NMF will be personalized for each user examined and will relate to that part of the social network that corresponds to the user's neighbors in question [20, 22, 23]. The most important and original feature of the proposed methodology is the introduction of ERGMs in the factorization process to mitigate the dominant local logic of the NMF, which focuses on the level of acne and considers each one independent of the others, to achieve the extracting latent factors that describe the placement of social network members in two or more communities and the explicit disclosure of cases of malicious use.

## 3. Proposed Methodology

Nonnegative factorization [24] of arrays is part of a broader family of dimensional techniques, which attempt to construct a partial representation of very high-dimensional data by projecting them into a lower-dimensional space. The difference in NMF from other methods is the limitation of the nonnegativity of the elements of the generated tables, which allows a better interpretation of the result. In the specific case we are considering, let *A* ∈ *R*_+_^*n*×*n*^ be a table adjacent to a graph of *n* nodes. We want to factorize it into two nonnegative arrays: the basis matrix *W* ∈ *R*_+_^*n*×*r*^ and the coefficient matrix *H* ∈ *R*_+_^*r*×*n*^, so that [25–27]:

(1) $$\begin{array}{c} A\approx Ae\equiv WH, \end{array}$$

where *r* is the number of communities and *r*≪*n*.

The purpose of the NMF is to calculate the elements of *W* and *H* so that their product is as “close” as possible to *A*, with proximity being measured by some distance function. More strictly, nonnegative table factorization is the following combination optimization problem (which in this case is described as a minimization problem) [28, 29]:

(2) $$\begin{array}{c} \underset{W,H}{\min},D\left(A\left\|WH\right.\right),\\ \text{Subject to,}W\geq 0,\;H\geq 0\text{,} \end{array}$$

where *D*(·*||*·) is a distance function of arrays *A* and *WH*. In general, the problem of minimizing the function *D* is NP-hard, for which additionally no convex formulations are known that would lead to finding the total minimum of *D* concerning both tables *W* and *H* at the same time. Although the optimization of *D* is non-convex for both arrays simultaneously, it is nevertheless convex for each of the two arrays separately; i.e., keeping unchanged, e.g., *W*, the problem becomes convex for *H* (and vice versa) [30].

To create an immediate and accurate forecasting process, we use the Bayesian NMF and the benefits through the retrospective allocation. The Bayesian NMF is a subcategory of the probabilistic NMF, which approximates the parameters *W* and *H* using the classical relation of the Bayesian inference [31, 32]:

(3) $$\begin{array}{c} P\left(W,H,\Theta |A\right)\alpha P\left(A|W,H,\Theta \right),P\left(W,H|,\Theta \right),P\left(\Theta \right), \end{array}$$

where the base and coefficient tables are the parameters of the model and Θ is the space of the hyperparameters (which regulate the statistical behavior of the distribution from which the tables *W* and *H* are derived). To use the above relation, a necessary condition is to speculate on the statistical origin of the neighborhood table data and the product's table factors. The left part of the relation expresses the a posteriori probability that the model parameters receive a specified value based on the specific data. In contrast, the first term of the product of the right part of the same relation expresses the likelihood of the model, the next is the a priori probability of model parameters for the specific hyperparameters, and finally, the last term is the likelihood of occurrence of the hyperparameters themselves. A careful selection of the probability function and the ex ante probability can result in an algorithm that exhibits better and faster convergence [33, 34]. Thus, the model's parameters must be observed, and based on this observation, the probability distribution that best expresses their statistical properties must be selected. Then, after the probability formation has been clarified, the appropriate pre-possibility for the area of the hyperparameters is set. To choose the proper a priori probability and probability function, factorization is performed, so the arrangement of the edges between the nodes is expected to show star-type phenomena, i.e., some few nodes with a large number of tangent edges and many nodes with a small number of tangent edges [35–37].

Therefore, there are many “open” triangles, and the most suitable ERGM for the occasion is the 2-star model, which is calculated as [34, 38, 39]follows:

(4) $$\begin{array}{c} H=\theta m\left(G\right)+\tau s\left(G\right),\\ m\left(G\right)=\sum_{i=1}^{n}\sum_{j=1}^{n}a_{i,j},s\left(G\right)=\sum_{i=1}^{n}\sum_{j=1}^{n}a_{i,j}\sum_{k=1\left(\neq j\right)}^{n-1}a_{ik}, \end{array}$$

where *a*_*ij*_ is the element of the graph adjacent table *A* (with value 1 if there is an edge between nodes *i* and *j*, and 0 otherwise), *m*(*G*) is the network statistic that models the number of edges of the graph (whose influence is controlled by the hyperparameter *i*), and *s*(*G*) is the corresponding magnitude for the number of 2 stars (whose influence is controlled by the hyperparameter *t,* respectively). It should also be noted that in this case, the graph is nondirectional (i.e., for the *i* and *j* elements of the neighborhood table, the equation *a*_*ij*_ = *a*_*ji*_ applies). Since there is no detailed solution for the model described, we must resort to approximate techniques for estimating the value of the hyperparameters *θ* and *τ*. As a first step, we rewrite the network statistics as a function, not of the edges, but the degree of *k*_*i*_ each node [33, 39, 40]:

(5) $$\begin{array}{c} m\left(G\right)=\frac{1}{2}\sum_{i=1}^{n}k_{i},s\left(G\right)=\frac{1}{2}\sum_{i=1}^{n}k_{i}\left(k_{i}-1\right)=\frac{1}{2}\sum_{i=1}^{n}k_{i}^{2}-m\left(G\right). \end{array}$$

Then, the Hamiltonian is derived as follows:

(6) $$\begin{array}{c} H=\theta m\left(G\right)+\tau s\left(G\right)=\frac{\tau }{2}\sum_{i=1}^{n}k_{i}^{2}+\frac{\theta -\tau }{2}\sum_{i=1}^{n}k_{i}. \end{array}$$

To facilitate the following calculations, the hyperparameters *θ* and *τ* are replaced by the auxiliary hyperparameters *J* and *B*, which are defined as follows:

(7) $$\begin{array}{c} J=\frac{\left(n-1\right)\tau }{2},\;B=\frac{\tau -\theta }{2}, \end{array}$$

so the Hamiltonian takes its final form:

(8) $$\begin{array}{c} H=\frac{J}{n-1}\sum_{i=1}^{n}{\left(k_{i}\right)}^{2}+B\sum_{i=1}^{n}k_{i}. \end{array}$$

Comparing the equations, we observe that they express the same ERGM [41], i.e., the 2-star model, using different network statistics. The free energy of the model described by the Hamiltonian is calculated as follows [40, 42, 43]:

(9) $$\begin{array}{c} F=-n\left(n-1\right)J{\left(\phi_{0}\right)}^{2}+\frac{1}{2}n\left(n-1\right)\ln\left(1+e^{4J\phi_{0}+2Β}\right)+\frac{n}{2}\ln\left[\left(n-1\right)J\right]-\frac{n}{2}\ln4\pi , \end{array}$$

where *φ*_*0*_ is the solution of the mean field approach given as follows:

(10) $$\begin{array}{c} \phi_{0}=\frac{1}{2}\left[\tanh\left(2J\phi_{0}+B\right)\mathrm{+1}\right]. \end{array}$$

The partial derivative of the free energy of the model for the hyperparameter *B* is equal to the expected value of the sum of the degrees of the nodes:

(11) $$\begin{array}{c} \langle \overset{n}{\underset{i=1}{\sum }}k_{i}\rangle =\frac{\partial F}{\partial B}\Rightarrow \overset{n}{\underset{i=1}{\sum }}\langle k_{i}\rangle =\frac{\partial F}{\partial B}. \end{array}$$

By approximating the expected value of the degree of each node 〈*k*_*i*_〉 with its most probable/expected value, i.e., the predicted value 〈*k*_*i*_〉 of the degree of all nodes, we have [44] the following:

(12) $$\begin{array}{c} \sum_{i=1}^{n}\langle k_{i}\rangle =\frac{\partial F}{\partial B}\Rightarrow n\langle k\rangle =\frac{\partial F}{\partial B}\Rightarrow \langle k\rangle =\frac{1}{n}\frac{\partial F}{\partial B}\Rightarrow \\ \langle k\rangle =\left(n-1\right)\frac{e^{4J\phi_{0}+2B}}{1+e^{4.J\phi_{0}+2B}}=\left(n-1\right)\frac{1}{2}\left[\tanh\left(2J\phi_{0}+B\right)\right]\\ \langle k\rangle =\left(n-1\right)\phi_{0}\Rightarrow \phi_{0}=\frac{n-1}{\langle k\rangle }, \end{array}$$

making *φ*_0_ equal to:

(13) $$\begin{array}{c} \phi_{0}=\frac{\langle k\rangle }{n-1},\;\phi_{0}\in \left(0,1\right). \end{array}$$

Similarly, the partial derivative of free energy for the auxiliary hyperparameter *J*/*n* − 1 is equal to the expected value of the sum of the squares of the degrees of the nodes [45]:

(14) $$\begin{array}{c} \langle \sum_{i=1}^{n}k_{i}^{2}\rangle =\sum_{i=1}^{n}\langle k_{i}^{2}\rangle =\frac{\partial F}{\partial \left(J/n-1\right)}. \end{array}$$

So, the expected value of the square of the degree of a node 〈*k*_*i*_^2^〉 is approximated by its most probable/expected value, that is, the expected value of the square of the degree of all nodes [28, 34, 46]:

(15) $$\begin{array}{c} \langle \sum_{i=1}^{n}k_{i}^{2}\rangle =\sum_{i=1}^{n}\langle k_{i}^{2}\rangle =\frac{\partial F}{\partial \left(J/n-1\right)}\Rightarrow n\langle k^{2}\rangle =\left(n-1\right)\frac{\partial F}{\partial J}\Rightarrow \langle K^{2}\rangle =\frac{n-1}{n}\frac{\partial F}{\partial J}\Rightarrow \\ \langle k^{2}\rangle =-{\left(n-1\right)}^{2}{\left(\phi_{0}\right)}^{2}+2{\left(n-1\right)}^{2}\frac{e^{4,J\phi_{0}\mathrm{+2}Β}}{1+e^{4J\phi_{0}\mathrm{+2}Β}}+\frac{1}{2}\left(n-1\right)\frac{1}{J}\Rightarrow \\ \langle k^{2}\rangle =-{\left(n-1\right)}^{2}{\left(\phi_{0}\right)}^{2}+2{\left(n-1\right)}^{2}\frac{1}{2}\left[\tanh\left(2J\phi_{0}+Β\right)\mathrm{+1}\right]+\frac{1}{2}\left(n-1\right)\frac{1}{J}\Rightarrow \\ \langle k^{2}\rangle =-{\left(n-1\right)}^{2}{\left(\phi_{0}\right)}^{2}+2{\left(n-1\right)}^{2}\phi_{0}+\frac{1}{2}\left(n-1\right)\frac{1}{J}. \end{array}$$

So:

(16) $$\begin{array}{c} \langle k^{2}\rangle =\langle -k^{2}\rangle +2\left(n-1\right)\langle k\rangle +\frac{1}{2}\left(n-1\right)\frac{1}{J}\Rightarrow \\ \frac{1}{2}\left(n-1\right)\frac{1}{J}=\langle k^{2}\rangle +{\langle k\rangle }^{2}-2\left(n-1\right)\langle k\rangle \Rightarrow \\ J=\frac{\left(n-1\right)}{2\left(\langle k^{2}\rangle +\langle k^{2}\rangle -2\left(n-1\right)\langle k\rangle \right)}. \end{array}$$

So:

(17) $$\begin{array}{c} 2\phi_{0}-1=\tanh\left(2J\phi_{0}+B\right)\Rightarrow 2J\phi_{0}+B=\tanh^{-1}\left(2\phi_{0}-1\right)\Rightarrow \\ B=\tanh^{-1}\left(2\phi_{0}-1\right)-2J\phi_{0}. \end{array}$$

Given the following identity, we have the following:

(18) $$\begin{array}{c} \tan^{-1}\left(x\right)=\frac{1}{2}\ln\frac{1+x}{1-x},\;\left|x\right|<1. \end{array}$$

So, it turns out:

(19) $$\begin{array}{c} B=\frac{1}{2}\ln\;\phi_{0}-\frac{1}{\langle k^{2}\rangle +{\langle k\rangle }^{2}-2\left(n-1\right)\langle k\rangle }. \end{array}$$

Therefore, the above equations fully describe the 2-star model in terms of the mean field. Given the original Hamiltonian of the solvable 2-star model, then the following relation is true [36, 40]:

(20) $$\begin{array}{c} H=-\frac{J}{n-1}\sum_{i=1}^{n}k_{2}^{i}+B\sum_{i=1}^{n}k_{i}=\frac{\partial F}{\partial J}J+\frac{\partial F}{\partial B}B=\nabla F. \end{array}$$

So, the Hamiltonian of the model is equal to the gradient of the free energy, and by substituting the values of *B* and *J,* we have [44]the following:

(21) $$\begin{array}{c} H=\nabla F=\frac{\partial F}{\partial J}J+\frac{\partial F}{\partial B}B\\ =-n\left(n-1\right)J{\left(\phi_{0}\right)}^{2}\mathrm{+2}n\left(n-1\right)\phi_{0}+\frac{n}{2}+n\left(n-1\right)B\phi_{0}. \end{array}$$

Therefore:

(22) $$\begin{array}{c} H=-n\left(n-1\right)J{\left(\phi_{0}\right)}^{2}+2n\left(n-1\right)J\phi_{0}+n\left(n-1\right)B\phi_{0}\\ =n\left(n-1\right)\phi_{0}\left[-J\phi_{0}\mathrm{+2}J+Β\right]\\ =n\langle k\rangle \left[-J\phi_{0}\mathrm{+2}J+\;\tanh^{-1}\left(2\phi_{0}-1\right)-2J\phi_{0}\right]\\ =\sum_{i=1}^{n}\langle k_{i}\rangle \left[2J-3J\phi_{0}+\;\tanh^{-1}\left(2\phi_{0}-1\right)\right]\\ =2\left[2J-3J\phi_{0}+\;\tanh^{\mathrm{-1}}\left(2\phi_{0}-1\right)\right]\frac{1}{2}\sum_{i=1}^{n}k_{i}\\ =\left[2\;\tanh^{-1}\left(2\phi_{0}-1\right)\mathrm{+2}\left(\mathrm{2-3}\phi_{0}\right)J\right]m\left(G\right). \end{array}$$

So, we end up:

(23) $$\begin{array}{c} H=\Theta m\left(G\right),\;\Theta =\ln\phi_{0}\mathrm{+2}\left(\mathrm{2-3}\phi_{0}\right)J. \end{array}$$

This is a significant conclusion for two reasons. Firstly, we were able to find an approximate solution in the 2-star model. Secondly, the specific solution we found can be easily integrated into the factorization process, thus displaying the entities that create the spam [14, 17].

Regarding the probability that models the local properties of the graph edges, in the probabilistic NMF we propose, the Poisson distribution optimizes the generalized Kullback–Leibler deviation. Thus, the following function greatly simplifies the required calculations [24, 44, 47]:

(24) $$\begin{array}{c} p\left(\overset{˜}{a_{ij}}|a_{ij}\right)\alpha \frac{\overset{˜}{a_{ij}}a_{ij}}{a_{ij}!}e^{-\overset{˜}{a_{ij}}}. \end{array}$$

Having chosen the probability distribution and the ex-probability, we can now use the classical relation of the Bayesian inference to approach the ex post probability that the parameters of our model (the elements of the table *Ae* = *WH*) take specific values, as well as the hyperparameters Θ of the model [48].

For the proposed model to succeed in imitating the actions of the human brain in a simplified way, the attention mechanism is used, which is also an attempt to implement the same measure of selective human concentration in some relevant things while ignoring some, respectively. This procedure allows for the particular treatment of different versions of the same situation and identifying events that significantly change the proposed control process. An abstract implementation related to the environment vector *c*_*i*_ for output *y*_*i*_ is generated using the weighted sum of the annotations so that [44]:

(25) $$\begin{array}{c} c_{i}=\sum_{j=1}^{T_{x}}\alpha_{ij}h_{j}. \end{array}$$

In the simplest case, the weights *α*_*ij*_ are calculated from a softmax function given by the following equation:

(26) $$\begin{array}{c} \alpha_{ij}=\frac{\exp\left(e_{ij}\right)}{\sum_{k=1}^{T_{x}}\exp\left(e_{ik}\right)}, \end{array}$$

where *e*_*ij*_ is the output rating of the feed mechanism described by function *a*, which attempts to record the alignment between the input to *j* and output to *i*.

So given that the element $\tilde{a_{ij}}$ results from the interior product of the *i*th row vector of the table *W* on the *j*th column vector of *H*, we arrive at the following relation [40, 44, 49]:

(27) $$\begin{array}{c} P\left(w_{i}^{T}h_{j}|a_{ij},\Theta \right)\alpha \frac{w_{i}^{T}h_{j}^{a_{ij}}}{a_{ij}!}e^{\left(\Theta -1\right)}w_{i}^{T}h_{j}. \end{array}$$

The slope of the equation for the vectors *w*_*i*_^*T*^*h*_*j*_ is calculated as follows:

(28) $$\begin{array}{c} \nabla D_{w_{i}^{T}}\left(a_{ij},w_{i}^{T},h_{j}\right)=\sum_{j=1}^{k}\left[-\frac{a_{ij}}{w_{i}^{T}h_{j}}h_{J}^{T}+\left(1-\Theta \right)h_{j}^{T}\right]\\ =-\frac{a_{i}^{T}}{{\tilde{a_{1}}}^{T}}H^{T}+\left(1-\Theta \right)e^{T}H^{T},\\ \nabla D_{hj}\left(a_{ij},W_{i}^{T},h_{j}\right)=\sum_{j=1}^{k}\left[-W_{i}^{T}+\frac{a_{ij}}{W_{i}^{T}h_{j}}+\left(1-\Theta \right)W_{i}^{T}\right]\\ =-W^{T}\frac{a_{i}^{T}}{{\tilde{a_{1}}}^{T}}+\left(1-\Theta \right)W^{T}e^{T}, \end{array}$$

while, respectively, they are renewed as follows:

(29) $$\begin{array}{c} {\left(W_{i}^{T}\right)}^{t+1}\leftarrow {\left(W_{i}^{T}\right)}^{\left(t\right)}{}^{\circ}\frac{\nabla D_{W_{1}^{T}}^{-}}{\nabla D_{W_{1}^{T}}^{-}}\Rightarrow {\left(W_{i}^{T}\right)}^{\left(t+1\right)}\leftarrow \frac{a_{1}^{T}/\tilde{a_{1}}H^{T}H^{T}}{\left(1-\Theta \right)e^{T}H^{T}}\\ h_{j}^{\left(t+1\right)}\leftarrow h_{j}^{\left(t\right)}{}^{\circ}\frac{\nabla D_{h_{j}}^{-}}{\nabla D_{h_{j}}^{+}}\Rightarrow h_{j}^{\left(t+1\right)}\leftarrow h_{j}^{\left(t\right)}{}^{\circ}\frac{Wa_{1}^{T}/\tilde{a_{1}}}{\left(1-\Theta \right)W^{T}e^{T}}, \end{array}$$

resulting in the following information rules for the base tables and coefficients *W* and *H*:

(30) $$\begin{array}{c} W^{\left(t+1\right)}\leftarrow W^{\left(t\right)}{}^{\circ}\frac{A/WHH^{T}}{\left(1-\Theta \right)E^{T}H^{T}},\\ H^{\left(t+1\right)}\leftarrow H^{\left(t\right)}{}^{\circ}\frac{W^{T}A/WH}{\left(1-\Theta \right)W^{T}E}. \end{array}$$

Thus, in combination with the deterministic calculation of Θ, we have the following:

(31) $$\begin{array}{c} 1-\Theta >0\Rightarrow \Theta <1\Rightarrow \;\ln\phi_{0}+2\left(2-3\phi_{0}\right)J<1. \end{array}$$

In conclusion, all social networks are far from being characterized as cliques, so the value of connectivity *φ*_0_ is much lower than unity and closer to zero (i.e., *φ*_0_ ≪ 1 applies). Therefore, the term ln  *φ*_0_ takes very small (negative) values. On the other hand, the hyperparameter *J* is positive (its use in the Hubbard–Stratonovich transform imposes its nonnegativity), so the second term of the sum is positive (it is an exact mathematical transformation that is used to convert a particle theory into its respective field theory by linearizing the density operator in the many-body interaction term of the Hamiltonian and introducing an auxiliary scalar field [50]). Thus, in social network graphs, the effect of the term ln  *φ*_0_ is stronger in shaping the final value of the hyperparameter Θ, so the above relation applies to all networks where the original hypotheses investigated apply. The contribution of ex ante probability to factorization (more specifically of ERGMs) and its contribution to the aggregation of members of a user's network to generate recommendations are to be quantified in the following experimental process.

## 4. Scenarios and Results

To model an email referral system, this study uses user actions about activity and the likelihood that a node will engage in abnormal behavior related to the spread of spam. The application is used based on a case study applied in the environment of educational organizations. Instead of classifying two classes into junk and desirable, we treat the problem as multi-category classification in which each class is a user recommendation action in an email. The most common activities are reading, replying, saving, waiting, deleting from the mailing list, terminating, including junk mail filters, and deleting. As part of a collaborative social filtering system, the previously described algorithm was applied to two different datasets containing user actions in mail messages and information about their social relationships.

The first collection is small and consists of usage data and, more specifically, the relevant statistics of a user's traffic to an educational podcast service. On the other hand, the second collection is medium-sized and contains educational material evaluations on an online learning platform on a five-point scale. Despite their differences, the two datasets are highly sparse and show the characteristics of free-scale networks regarding the number of ratings they contain and the distribution of social network nodes (most edges fall on a few nodes, while most nodes touch a few edges). A user's ratings are extracted from the data collection and divided into two distinct sets: training and testing. The test set is then repositioned in the data collection. In the next step, the algorithms generate recommendations, and a list of objects is returned as output, compared with the data in the test set. The whole process is repeated five times for a list size of 5 to 25 items. For the generated recommendations to make sense, in each iteration of the experimental protocol, only users are selected who have evaluated at least twice the number of objects from the respective list size. Several memorandum collaborative systems were implemented to assess the quality of the recommendations produced in different environments.

The levels of the metrics refer to the average of the respective values, while the similarity function used was the logarithm of the probability ratio of the data. Local-level trust metrics were also examined, namely the MoleTrust 1 algorithm (which considers only the users with whom the user in question is directly associated with acne) and the MoleTrust 2 (which also finds the neighbors of the user in question). TidalTrust was also implemented, which calculates user similarity based on the shortest paths between the user in question and all other users on the same connected component. The comparison also includes an algorithm that calculates each user's reputation on the social network and, more specifically, TrustWalker, which takes a random walk on the graph, selecting its next step uniformly at random. The random walk starts from the user in question, and when it reaches its stationary distribution, the nodes with the highest probability are returned as more similar. Finally, the user network clustering methodology was tested to estimate the relative performance of ERGMs in the position of ex ante probability. The configuration includes the Bayesian NMF algorithm using the Poisson distribution in place of the likelihood. The method of filtering the neighbors that were applied was that of the nearest-N; i.e., in producing the recommendations, only the *N* closest neighbors are taken into account. The value of parameter *N* was set to 5 after a series of verification experiments in which it was found that for values of *N* less than 5, the results of the metrics were unstable. In contrast, the results were lower for values of *N* greater than 5. It should be noted, however, that the relative classification of the algorithms remains constant, regardless of the value of *N* [20, 23, 31, 44, 51].

A first observation of the results is that the competing algorithms perform significantly lower performance than the proposed one. This behavior is attributed to the fact that the proposed methodology's interaction between users and objects is deemed valid and can be the basis for a possible recommendation. Another interesting point is that the density ratio between the two datasets is reflected almost linearly in the individual results achieved by the different systems. An equally important observation is that each user's network is a good source of information for making recommendations in sparse datasets, clearly superior to traditional collaborative methods. As the level of data dilution increases, the efficiency of conventional collaborative recommendation algorithms decreases, as there are fewer and fewer users whose object ratings match. In this case, social algorithms “unfold” all their dynamics. The results prove the apparent superiority of the social algorithms that perform a local search on all metrics. It is also worth noting that all three algorithms based on local social network search show similar results even though they explore each user's neighborhood at a different depth.

Nevertheless, they do not perform well in either dataset. The above observation concludes that basing the recommendations exclusively on the social network's most popular (or the most frequently visited) nodes does not guarantee similarity in preferences. Finally, the proposed methodology seems to achieve the best results compared with the local and the full search on the social network. Placing the nodes that are part of a user's network in overlapping clusters leads to complete analysis of users' proximity to the network, especially when compared to the basic assumptions made by other algorithms. The addition of ERGMs further improves this analysis in place of the ex ante probability. In this way, the central view given to each acne by the Bayesian NMF is addressed, i.e., by introducing structural features of the graph in the process.

## 5. Conclusions

The consequent increase in the popularity of online educational resources, combined with this lack of preparedness, has made the education sector an ideal target for digital phishing attacks. The detection of spam and the timely assessment of these threats allow the detection of events that can significantly mitigate the effects of organized cyberattacks. An advanced deep attention collaborative filter was presented to ensure the educational processes and the protection of the educational system. It is a specialized application of intelligent techniques where the spam problem is examined as a social graph to identify harmful communities. Using a deep attention mechanism, the methodology becomes personalized for each user, while critical, innovative optimization processes help evaluate social graphs.

This methodology is being tested with great success in implementing mail protection systems for educational organizations based on the integration of mnemonic, social, and collaborative recommendation systems. The results obtained show a steady improvement of all performance metrics compared with all comparable implementations. This improvement is attributed to the unique filtering capabilities of the proposed methodology. Instead of summing up many users, it tries to discover patterns in their social behavior by (overlapping) grouping them into regions. This process bears similarities to how overlapping finding commonalities algorithms are operating.

A possible research direction would be to include factorizing more complex network features, such as triangles. In this case, however, the model calculations become pretty complicated. It is not easy to derive an approximate solution similar to the one presented, so the implementation with advanced equipment such as GPU or TPU should be investigated. Accordingly, a more generalized approach to model estimation should be explored as to whether they would achieve better results and greater generalization in community evaluation. Finally, adopting post-hybrid methods of automatic optimization of communities is considered very important for the further development and use of the methodology.

## Data Availability

The data are available on reasonable request.
